# The Lateral Line Facilitates Rapid Responses in Accelerating Fish Schools

**DOI:** 10.1093/icb/icag151

**Published:** 2026-08-18

**Authors:** Ashley N Peterson, Ji Zhou, Rajat Mittal, Matthew J McHenry

**Affiliations:** Department of Ecology and Evolutionary Biology, University of California, 321 Steinhaus Hall, Irvine, CA 92697, USA; Mechanical Engineering, Johns Hopkins University, Baltimore,MD 21218, USA; Mechanical Engineering, Johns Hopkins University, Baltimore,MD 21218, USA; Department of Ecology and Evolutionary Biology, University of California, 321 Steinhaus Hall, Irvine, CA 92697, USA

## Abstract

The collective motion of a fish school emerges from communication between its members, yet the sensory mechanisms mediating this communication remain largely unclear. While the visual system is essential for schooling in a diversity of species, the processing of visual stimuli is substantially slower than the mechanosensory lateral-line system. Through experimental manipulation, previous experiments found only modest contributions by the lateral line on time-averaged schooling kinematics, but substantial changes on the network structure of information sharing. To resolve this apparent discrepancy, we combined hydrodynamic modeling with kinematic and network analysis of schools of 60 rummy-nose tetra (*Petitella bleheri*). These fish exhibit intermittent motion that requires individuals to respond rapidly to their neighbors during periods of acceleration and deceleration to maintain a cohesive school. Through an analysis of the pairwise cross-correlation of speed during accelerations, we found that fish capable of sensing the flow of their neighbors responded with a latency that was one-third less than that of schools of fish with a compromised lateral line. In addition, flow-sensing schools exhibited higher mutual information (MI) and a more efficient and uniform communication network. No differences in response latency or MI were observed during periods of deceleration. These results demonstrate that the lateral line serves not as a redundant channel to vision, but as a fast sensory pathway that tightens temporal coupling between neighbors during the most rapid motion of fish schools.

## Introduction

Fish schooling represents one of the most tractable systems for studying collective behavior. The study of schooling has shown how complex group patterns emerge from simple local rules without centralized control (Aoki 1982; Partridge 1982; Heydari and Kanso 2021) and how information propagates through groups to enable collective decision-making (Couzin et al. 2005). Beyond coordination, schooling confers tangible benefits to individuals by reducing predation risk through dilution and predator confusion (Pitcher 1993; Wood and Ackland 2007; Cooper and Blumstein 2015), enhancing foraging success (Pitcher and Magurran 1982; Pitcher et al. 1982; Godin et al. 1988; Day et al. 2001), and reducing the energetic cost of swimming (Svendsen et al. 2003; Hemelrijk et al. 2015; Marras et al. 2015; Ashraf et al. 2017; Seo and Mittal 2022; Zhang and Lauder 2024). Schools also exhibit phase transitions between collective states, suggesting that the underlying dynamics are rich and nonlinear (Tunstrøm et al. 2013; Harpaz et al. 2017, 2022). Yet despite this wealth of knowledge about group-level outcomes, it remains unclear how such cohesive motion depends on the sensory modalities of individual fish.

Vision and flow sensing serve as candidate modalities for mediating schooling due to their capacity for stimulating rapid responses. The lateral-line system allows fish to respond to flow in less than 10 ms, whereas the latency to visual stimuli is generally 20*×* greater (Coombs et al. 1989, 2014; Liu and Fetcho 1999). This difference reflects the substantial neuronal processing required in the retina and optic tectum for vision, whereas mechanosensory systems such as the lateral line require fewer synapses from stimulus to muscular action (Burgess and Shaw 1981; Jagger 1992; McHenry and Peterson 2025). Vision has the advantage of allowing fish to sense their neighbors at a greater range, and with greater spatial acuity, than the lateral-line system can offer. It is therefore possible that these two modalities offer complementary sources of information to a schooling fish.

Experimental manipulations have offered valuable insight into the roles of vision and flow sensing in schooling. A diversity of species cannot maintain group cohesion in the dark, suggesting that visual cues are necessary for the behavior (Hunter 1968; Ali 2001; Torisawa et al. 2007; Kowalko et al. 2013; Schulze et al. 2018; Ginnaw et al. 2020; McKee et al. 2020). Yet, individual Pollock can hold position within a school when blinded (Pitcher et al. 1976), and this ability is lost only when the lateral-line afferent nerve is additionally severed (Pitcher et al. 1976; Partridge and Pitcher 1980). This result underscores a role of flow sensing as at least a compensatory channel of communication. The lateral line has since been examined more broadly through chemical ablation experiments (Partridge and Pitcher 1980; Faucher et al. 2010; McKee et al. 2020; Peterson et al. 2024; Tidswell et al. 2024), revealing that schooling does not require the lateral line, provided the fish can see (McKee et al. 2020; Peterson et al. 2024; Tidswell et al. 2024). In rummy-nose tetras, ablation caused only modest changes to group kinematics: the nearest-neighbor distance (NND, see Table 1 for a list of symbols) increased by roughly 15% and polarization decreased by a similar margin (Peterson et al. 2024). These results suggest that vision is the primary mediator of schooling and that flow sensing merely offers a modest enhancement to vision. However, population-level averages of conventional schooling metrics may obscure the role of flow sensing in the temporal dynamics of a school, particularly for rapid neighbor-to-neighbor communication.

Information metrics present the opportunity to measure how communication propagates through a school with unsteady motion. In social animals, similarity in the temporal pattern of kinematic variables (e.g., speed and/or heading) infers communication and motivation for the animals to move together (Schaerf et al. 2017; Ward et al. 2018; Kent et al. 2019). Such similarity has been successfully quantified through an application of Shannon entropy to measurements of swimming kinematics in schooling fish (Swanson et al. 2026; Peterson and McHenry 2026). One such metric, mutual information (MI), yields a measure of the information flow through the individuals in a network (Shannon and Weaver 1963; Shannon 1948). Measures of MI can reflect responses of fish to one another or to a common stimulus, such as an obstacle or oncoming flow. Because it does not require an estimate of latency, MI can provide a robust measure of similarity in motion that may serve as the quantitative basis of a social network. By treating individual fish as nodes and MI as edges in a network, measures of network properties can reveal information flow through a school (Berdahl et al. 2013; Rosenthal et al. 2015). Such analyses have shown how schools facilitate rapid information propagation, despite primarily local interactions (Handegard et al. 2012), and that network topology dynamically reorganizes during predator evasion maneuvers (Katz et al. 2011). Furthermore, network-based metrics have enabled measurements of leadership and influence within schools. For example, Strandburg-Peshkin et al. (2013) showed that individuals in positions of high centrality play disproportionate roles in coordinating collective motion, while Rodriguez-Santiago et al. (2020) found that the relationship between centrality and social influence depends on behavioral context.

The present study revisits prior experiments on flow sensing with a detailed examination of its mechanistic basis. Prior work on the sensorimotor basis of schooling showed that, within schools of 15 to 60 fish, flow sensing had the largest influence on kinematics and the social network of the largest schools (Peterson et al. 2024). Here, we hypothesized that the lateral-line system serves not merely as a supplemental modality to vision, but instead facilitates rapid coordination between individuals within the school. Specifically, we predicted that flow sensing would generate more rapid responses to hydrodynamic signals from neighbors during strong collective accelerations. Furthermore, the loss of flow sensing was expected to reduce the efficiency and uniformity of the school’s communication network, reflecting an inability to respond rapidly to neighboring fish. To test these predictions, we applied a combination of hydrodynamic modeling and kinematic analysis to resolve the temporal similarities in swimming speed and heading and to model the flows that facilitate communication during schooling. We analyzed the schooling kinematics and network properties from six schools of 60 rummy-nose tetra (*Petitella bleheri*, formerly *Petitella rhodostoma*) from Peterson et al. (2024). This species exhibits intermittent motion at the individual and school levels, with continuous oscillations in speed. The fish were either treated with neomycin sulfate to compromise the lateral line (*N=3*) or were untreated and therefore capable of sensing water flow (*N=3*; see Peterson et al. 2024 for methodology). Our kinematic analysis examined how temporal variation in network properties varied with the position of fish within the arena and during accelerations and decelerations. We modeled the wakes generated by fish in these experiments using a recently published model for the hydrodynamics of fish schools in a cylindrical arena (Zhou et al. 2025) that replicates the conditions of the experiments in Peterson et al. (2024). This combination of approaches allowed us to address the role of flow sensing in the intermittent motion of fish schools.

## Analytical approach

### Data acquisition

In previous experiments, schools of rummy-nose tetra (*Petitella bleheri*; average body length [BL], 3.17 cm *±* 0.01 standard error, SE, sampled from 30 individuals) were divided into two flow sensing treatment categories: those without the ability to sense flow (treated) and those with intact flow sensing (untreated). Each treatment category comprised three schools of 60 individuals, with fish randomly selected from a larger pool. Fish in the treated schools were exposed to an aminoglycoside antibiotic, neomycin sulfate, prior to experiments (990 $\mu \mathrm{M}$, determined by a preliminary assay; Peterson et al. 2024). Aminoglycosides damage the mechanosensory hair cells of the lateral-line system, compromising a fish’s ability to sense flow stimuli. The effects of aminoglycosides are dose- and time-dependent; although overexposure can damage the hair cells of the inner ear and result in hearing loss, we did not observe any adverse effects of the treatment (e.g. an inability to maintain equilibrium and distressed or erratic swimming behavior; Harris et al. 2003; Murakami et al. 2003; Faucher et al. 2008; Owens et al. 2009; Mekdara et al. 2022).

Schools were filmed from above while freely interacting in a large shallow (3 cm) still-water arena, constrained within a circular barrier (diameter 1.03 m) (Peterson et al. 2024). The arena was back-lit with infrared LEDs and illuminated from above with visible light. The body-center coordinates and heading of individuals were tracked from video recordings of the fish and were analyzed with a combination of open-source packages and custom software scripted in the Python language (v.3.10). Once the videos were preprocessed (see Peterson et al. 2024 for details), fish were tracked with TRex (Walter and Couzin 2021, Fig. 1A). Data analyses were performed in MATLAB (v2025a, Mathworks, Natick, MA, USA), including outlier identification and processing, as well as interpolation and smoothing of raw kinematic data. We performed a number of measurements of the individual motion and schooling kinematics from the position and heading of individual fish. This included metrics of spacing and similarity in heading by calculating the NND and the polarization parameter (Couzin et al. 2002). NND was calculated as the proximity between the center-of-body of each fish and its nearest neighbor for each video frame. The polarization parameter was used to determine the extent to which the headings of fish within a school are similar (a value of 1 indicates uniform alignment), calculated as follows:


(1) \begin{eqnarray*}
\begin{split} \rho =\frac{1}{n} \sqrt{ \left( \sum _{i=1}^{n} \sin \theta _i \right)^2 + \left( \sum _{i=1}^{n} \cos \theta _i \right)^2 }, \end{split}
\end{eqnarray*}

where *n* is the number of fish in a school and *θ* is the heading of an individual.

**Fig. 1:**
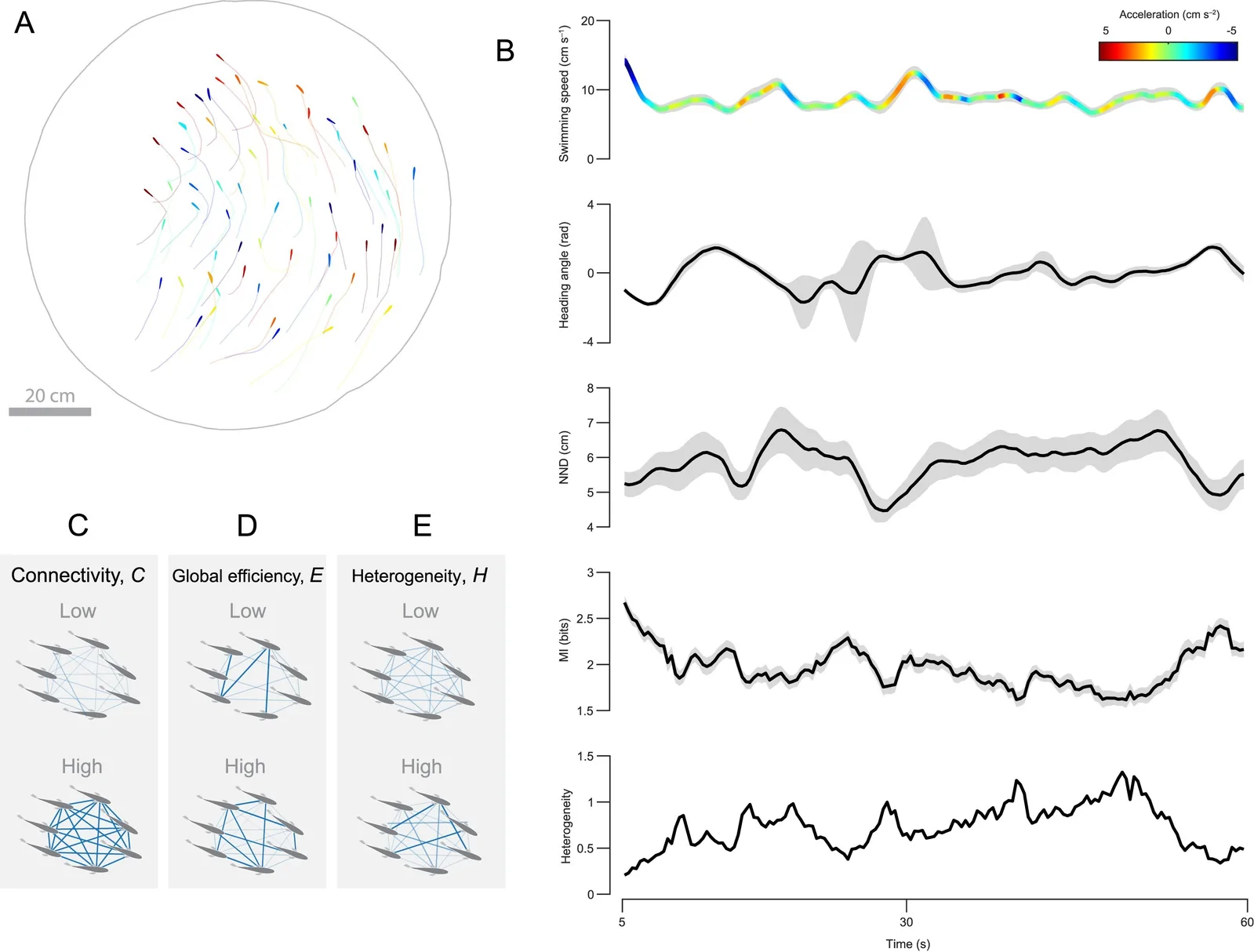
Measurements of schooling kinematics in rummy-nose tetras. (A) A representative video frame is depicted with each fish shown in a colored outline with trajectories from previous positions in time and the bounds of the experimental arena (in gray) for a school of 60 fish. (B) Representative kinematics and network metrics for a school. These include the mean values across the school for speed, heading, NND, and the MI between fish. The network heterogeneity (Equation 4) was calculated from the network of MI values for the school at each moment of time. In all time series panels, shading depicts the 95% CI of the data, with acceleration values overlaid on the speed curve. (C) Schematic illustrations of schooling network properties. These include connectivity (Equation 3), global efficiency (Equation 5), and heterogeneity (Equation 4), with edges (thicker lines indicate higher intensity) connecting the nodes (individual fish) within the network.

### Network analysis

We characterized similarity in heading and speed between all pairs of fish in a school using MI (Kraskov et al. 2004). MI was calculated over a sliding 2 s interval, with 0.25 s overlap between successive intervals. For each interval, the speed and heading of each fish were discretized into eight bins, where bin edges were set so that each bin contained an equal number of measurements across all fish. This resulted in 64 kinematic states (8 × 8) that captured the joint distribution of speed and heading. After scoring the proportion of measurements of speed and heading that occurred within the set kinematic bins for a pairwise interaction (for fish ‘a’ and fish ‘b’), we calculated the marginal probability (*p(a)* and *p(b)*) and joint probability distribution (*p(a, b)*). These measurements provide the basis for calculating MI, as follows (Kraskov et al. 2004):


(2) \begin{eqnarray*}
M(A; B) = \sum _{a \in A} \sum _{b \in B} p(a, b) \log _2 \left( \frac{p(a, b)}{p(a) p(b)} \right),
\end{eqnarray*}

where *M(A; B)* is the adjacency matrix of MI values between individuals.

We used MI to provide the basis of a communication network that allowed us to quantify network properties of each school (Fig. 1C). Individual fish were treated as nodes in this network with edges defined by pairwise MI values above a threshold value (MI$_\mathrm{thresh}$ = 2.11 bits). The number of edges associated with each node (above the MI threshold), known as the degree (deg(*i*), for the *i*th node), was used to approximate the number of individuals to which each fish’s movement resembles. Algebraic connectivity (*C*) characterizes the coherence of the network, defined as the second smallest eigenvalue ($\mathrm{eig}_2(\cdot )$) of the Laplacian matrix of the network.


(3) \begin{eqnarray*}
C = \mathrm{eig}_2(\Omega - M),
\end{eqnarray*}

where *Ω* is a diagonal matrix from the MI matrix ($\Omega _{i,i} = \sum _{j} M_{i,j}$). We also quantified variation in connectivity across individuals (network heterogeneity, *H*) as the coefficient of variation of node degree (Rodrigues 2019):


(4) \begin{eqnarray*}
H = \frac{\sigma _{\mathrm{deg}}}{\mu _{\mathrm{deg}}}.
\end{eqnarray*}

The distance between two nodes was characterized by the shortest path connecting them, or the minimum number of edges in series. To determine how efficiently information can propagate through the school, we calculated the global efficiency (*E*), the inverse of the mean shortest path length of all node pairs (Latora and Marchiori 2001).


(5) \begin{eqnarray*}
E_{\mathrm{glob}} = \frac{1}{n(n-1)} \sum _{i \ne j} \frac{1}{d_{i,j}},
\end{eqnarray*}

where $d_{i,j}$ is the shortest path length between nodes *i* and *j* of the MI pairwise matrix; pairs that are not connected (infinite $d_{i,j}$) contribute zero to the sum. Thus, when many pairs are connected by short paths, *E* is high.

### Latency analysis

To evaluate how delays in the responses of fish to one another affect the kinematics of schooling, we characterized oscillations in school speed over the course of each recording. Rummy-nose tetras swim intermittently, and this is reflected at the group level by accelerations and decelerations of the whole school. We averaged school speed with the same sliding interval (2 s duration, 0.25 s overlap) used to determine MI and then applied a custom peak-finding script (using the findpeaks function in MATLAB) to isolate peaks and troughs in speed. The identified periods of acceleration and deceleration spanned over 90% of the duration of all experiments and formed the time segments used for all subsequent analyses, including the evaluation of how flow sensing influenced schooling behavior.

**Fig. 2:**
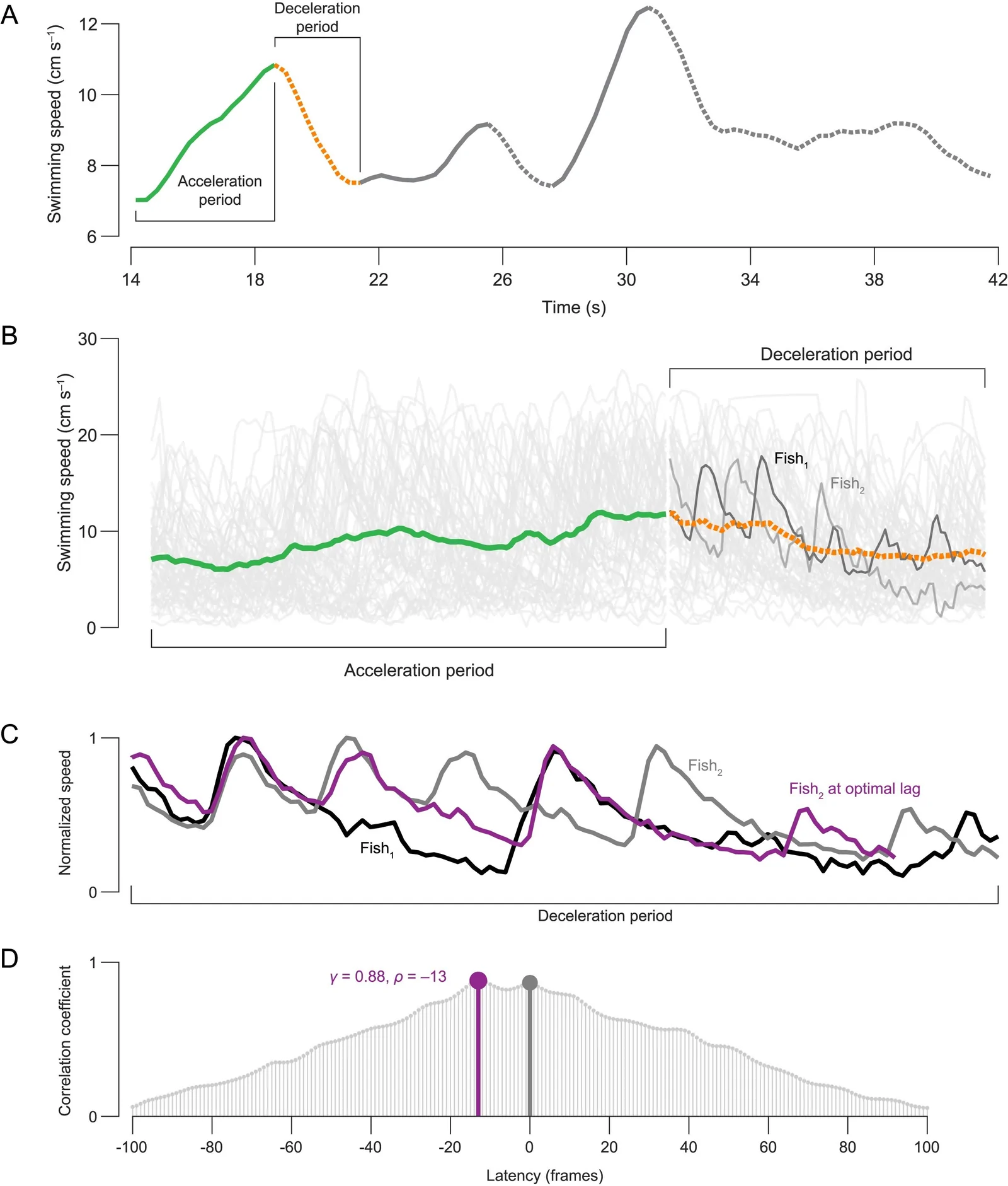
We evaluated the coordination and timing of pairwise fish speed with a cross-correlation analysis within a school of rummy-nose tetras. (A) Peaks and troughs in speed were used to isolate periods of acceleration (solid curves) from periods of deceleration (dashed curves) for a school. (B) For each segment, we performed a pairwise cross-correlation analysis of individual measurements of fish speed (gray curves) while the school accelerated (mean speed in green) and decelerated (mean speed in orange). Highlighted are traces for individuals: Fish$_{1}$ (in black) and Fish$_{2}$ (in dark gray) during deceleration. (C) Coordination and timing of swimming speed between individuals were determined by cross-correlation, which determines the latency that minimizes the difference between signals. Here, the latency of Fish$_{1}$ is subtracted from the timing of its values (in purple) in comparison with Fish$_{2}$. (D) The correlation coefficient values (in light gray) are maximal (in purple) for the latency that minimizes the difference in speed between the fish. A zero latency (dark gray) offers a lower correlation coefficient value.

We also evaluated how the intensity of acceleration and deceleration contributed to the timing and coordination of behavioral responses between fish. The magnitude of a behavioral period was determined based on the linear slope of school speed, a metric of mean acceleration during the period. Thus, absolute value of acceleration and deceleration periods greater than the population median value were considered “high” magnitude, while periods less than the median were categorized as “low” magnitude. To quantify temporal coupling in speed between individuals, we computed pairwise cross-correlations of frame-by-frame speed within each segment of acceleration or deceleration (Fig. 2A and B). For a given segment and fish pair *(i,j)*, we computed the discrete cross-correlation of the speed traces and identified the peak correlation coefficient ($\gamma _{i,j}$) and its associated latency ($\tau _{i,j}$) (Fig. 2C and D). Each qualifying fish pair was thus assigned a single *γ* and *|τ |* value per segment. Segment-level averages (e.g., *Γ* and $|\mathrm{T}|$ over all included pairs) were then used for statistical analyses of network properties. We also recorded the pairwise distance between fish for all video frames, from which we calculated the pairwise minimum distance (*d*) within a behavioral period as the minimum distance between pairs within a segment.

### Hydrodynamic modeling

To investigate how fish responded to flow cues during schooling, we applied the agent-based flow model of Zhou et al. (2025) to the recorded kinematics of schools with intact flow sensing (*N=3* schools). The model decomposed the flow speed sensed by each focal fish into two components: the potential flow generated by the displacement volume of a fish body and the vortices generated by each half tail-beat that form the chain-like vortex wake. The measured position and heading of each fish specified the location and orientation of both focal and neighboring fish. The model required steady swimming parameters, so the mean school speed measured previously was used for all three schools (2.88 BLs per second (BL/s), Peterson et al. 2024). Tail-beat frequency and amplitude were not measurable from the experimental videos due to the small number of pixels occupied by each fish; we therefore used values measured during steady swimming at a comparable speed in rummy-nose tetras from Ashraf et al. (2017, Fig. 3A and B; 8.41 Hz and 0.19 BL, respectively). These parameters defined the rate of vortex shedding and the potential flow generated by each fish. The flow speed sensed by each fish at each time step was determined by averaging speed contributions across six body-surface sensing points (Zhou et al. 2025).

**Fig. 3:**
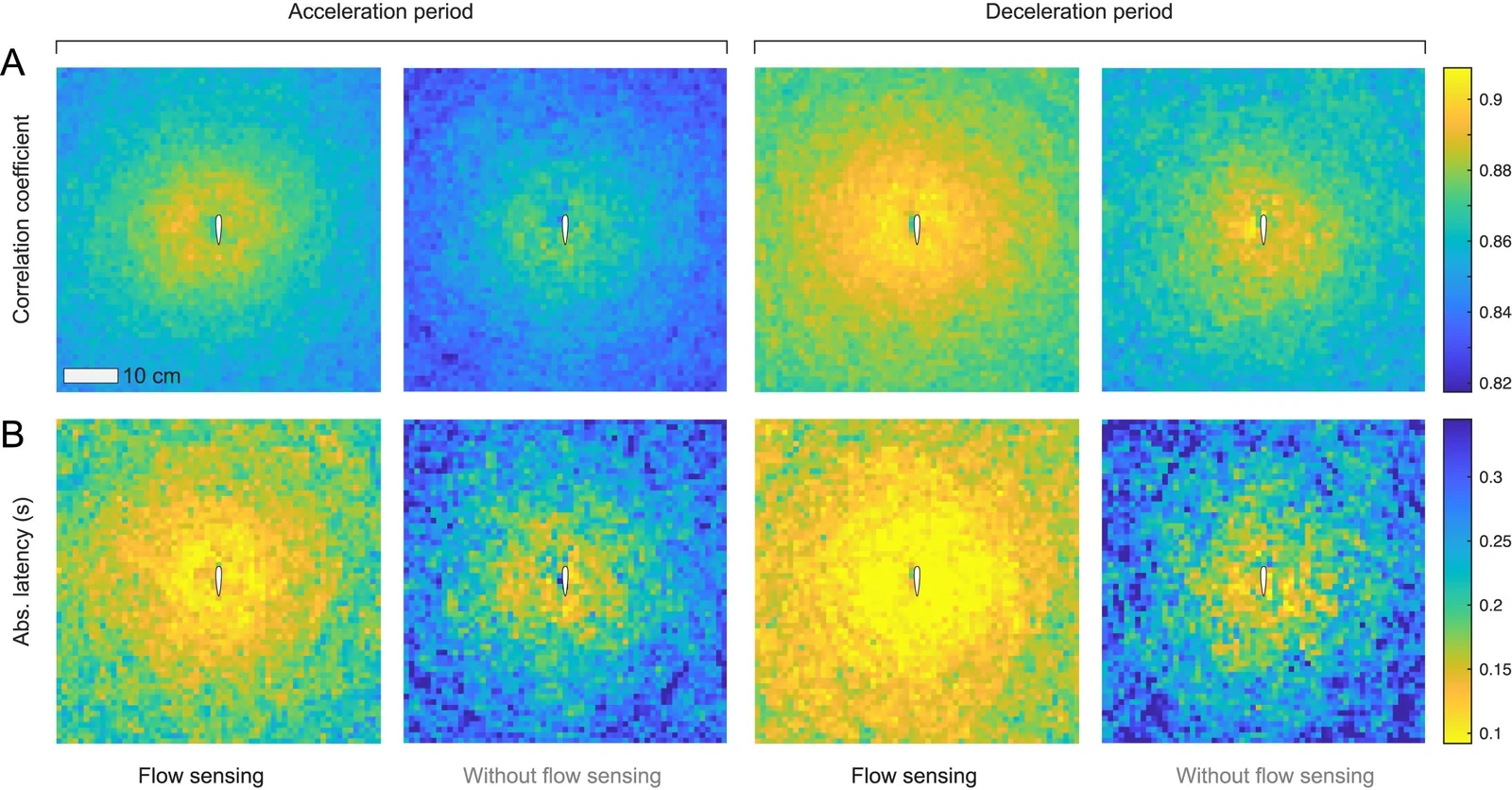
The average spatial distribution of correlation coefficients and the latency of speed changes between schooling rummy-nose tetras. Each colormap presents the mean values that correspond to neighboring fish at each position, across all experiments (*N* = 3, per treatment group: LLS present, LLS absent), for all fish, for all instants of time and are expressed with respect to the position and heading of the focal fish (shown in the center, oriented vertically, not to scale). (A) Warm coloration depicts overall higher coordination, indicated by higher correlation coefficient (*γ*) at close proximity to the focal fish, particularly during periods of deceleration. (B) Here, warmer colors depict shorter response latency *|τ |* in fish with flow sensing overall, particularly at close range to other fish.

We performed a cross-correlation between the flow speed, *u*, and fish swimming speed within each behavioral period. Unlike in the pairwise cross-correlations of fish speed, flow speed was always the reference signal. Therefore, the sign of the latency indicated whether changes in the speed of a fish followed changes in the flow speed it detected (negative latency) or preceded them (positive latency). This analysis provided a means to determine whether fish with flow sensing responded to (negative latency), or were unaffected by (positive latency), local hydrodynamic cues.

### Statistical analysis

We used linear mixed-effects models to test how the magnitude of collective speed changes, pairwise minimum distance, and the loss of flow sensing affected schooling kinematics and network properties. All statistical analyses were performed with the R scripting language, using the ‘lmerTest’ package (R Core Team 2024; Kuznetsova et al. 2017). The dependent variables consisted of the time-averaged values of each schooling metric within a behavioral period. We did not consider possible interactive effects between the variety of possible variables due to the necessary reduction in degrees of freedom and elevated model complexity. Statistical analysis evaluated the effect of treating the lateral line (*n = 3* schools of 60 fish), compared with the untreated (*n = 3*) schools. Analysis of cross-correlations for flow speed and swimming speed focused only on the untreated schools. The following statistical models were applied separately for acceleration or deceleration periods.

Our first statistical model considered the magnitude of an acceleration/deceleration period and the effect of the treatment upon the lateral line system (LLS) on each dependent variable (*Y*). This model is shown in Wilkinson notation (Wilkinson and Rogers 1973) as follows:


(6) \begin{eqnarray*}
Y \sim \mathrm{LLS} + m + (1|\mathrm{Exp}),
\end{eqnarray*}

where individual experiments were accounted for with random effects (Exp). Dependent variables included school speed, polarization, and properties of the social network (degree, global efficiency, heterogeneity, and connectivity).

We also tested how the proximity between fish during an acceleration/deceleration period and the loss of flow sensing affected pairwise coordination and information exchange. In this model, pairwise minimum distance (*d*) and whether the LLS was compromised served as effects on each dependent variable (*Y*). Here, dependent variables were MI, metrics of coordination (*γ*, the correlation coefficient), and the response latency (*|τ |*, the abs. pairwise response latency):


(7) \begin{eqnarray*}
Y &\sim & d + \mathrm{LLS} + (1|\mathrm{Exp}) + (1|\mathrm{Exp}:\mathrm{Seg}) \\&&+ (1|\mathrm{Exp}:\mathrm{Pair}),
\end{eqnarray*}

where intercepts for grouping factors (e.g., experiment, repeated behavioral segments (Seg), and pairwise (Pair) interactions) were included as random effects. We then examined whether the magnitude of a behavioral period ($m_\mathrm{high}$ or $m_\mathrm{low}$) influenced the dependent variable, *Y*, within each treatment group (LLS present; LLS absent), while accounting for the additive effect of pairwise distance (*d*). Here, the dependent variables are the same as in Equation (7):


(8) \begin{eqnarray*}
Y &\sim & d + m + (1|\mathrm{Exp}) + (1|\mathrm{Exp}:\mathrm{Seg}) \\&&+ (1|\mathrm{Exp}:\mathrm{Pair}),
\end{eqnarray*}

where random intercepts for grouping factors were included as random effects. Similarly, we examined whether there was an explicit effect of flow sensing on each dependent variable, *Y*, within behavioral periods of specific magnitudes ($m_{\mathrm{high}}$ or $m_{\mathrm{low}}$), while accounting for the additive effect of pairwise distance (*d*). Here, the dependent variables are the same as in Equations (7) and (8):


(9) \begin{eqnarray*}
Y &\sim & d + \mathrm{LLS} + (1|\mathrm{Exp}) + (1|\mathrm{Exp}:\mathrm{Seg}) \\&& + (1|\mathrm{Exp}:\mathrm{Pair}),
\end{eqnarray*}

where random intercepts for grouping factors were included as random effects.

Lastly, we tested the effect of behavioral magnitude on each dependent variable (*Y*), which here are the flow cross-correlation coefficient and corresponding latency values:


(10) \begin{eqnarray*}
Y \sim m + (1|\mathrm{Exp}) + (1|\mathrm{Exp}:\mathrm{Seg}) + (1|\mathrm{Exp}:\mathrm{Fish}), \,\,\,
\end{eqnarray*}

where random intercepts for grouping factors (e.g., experiment, repeated behavioral segments, Seg, and individual fish, Fish, interactions) were included as random effects.

## Results and discussion

### Sensory modulation of collective kinematics

**Fig. 4:**
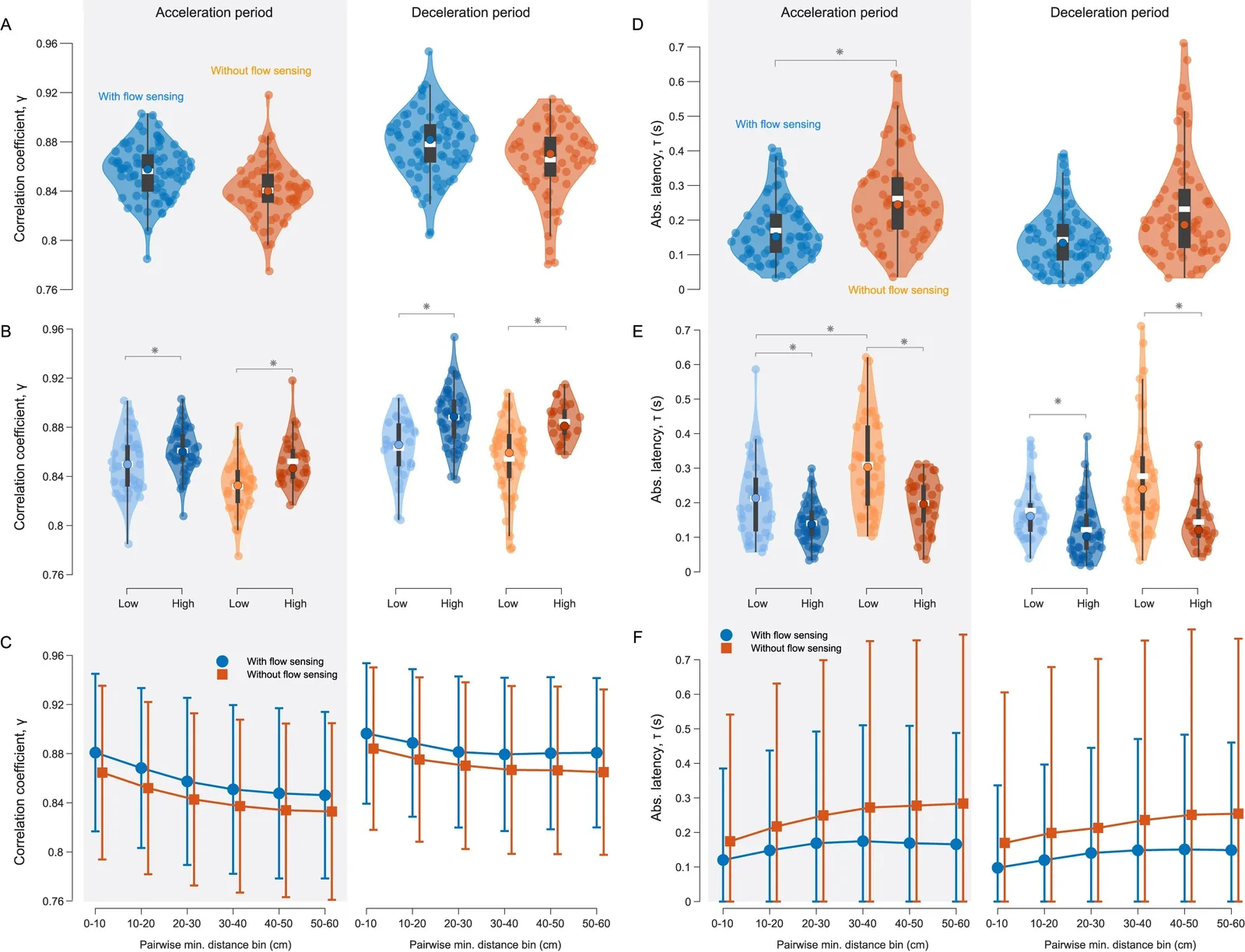
The results of a cross-correlation analysis evaluating coordination and latency in speed between individual rummy-nose tetras during periods of school acceleration and deceleration. (A and D) Violin plots depict the probability distributions of segment averages (transparent colored dots) of *γ* and *|τ |* within behavioral periods for schools that can sense flow (in blue) and those that cannot (in orange). (B and E) Violin plots depict the probability distributions of segment averages (transparent colored dots) within a behavioral period, subdivided by the magnitude of the behavior period. Light and dark shades of each color depict “low” and “high” magnitude, respectively. (A, B, D, and E) A box plot within each violin plot depicts the first and third quartiles of the data, the median (solid colored dot), and the mean value (white bar); whiskers show the range of the data. Connecting horizontal lines with an asterisk depict significant relationships between groups of data, and error bars depict *1±* standard deviation. (C and F) The effects of pairwise distance on (C) the correlation coefficient and (F) response latency for schools with flow sensing (in blue) and without flow sensing (in orange). Pairwise values of *γ* and *|τ |* were averaged within 10 cm pairwise minimum distance bins (circle and square markers). For display, we truncated distance bins to 60 cm. Error bars depict *1±* standard deviation. Asymmetrical error bars are shown in panel F, where SD was truncated to zero to accurately reflect the *|τ |* used in the present analysis.

Cross-correlation analysis (*γ*) and response latency (*|τ |*) provide a quantitative basis for evaluating the contribution of flow sensing to the coordination of schooling behavior. Since mechanosensory hair cells detect flow gradients on much shorter timescales (tens of milliseconds) than visual processing (hundreds of milliseconds), the LLS has the potential to tighten temporal coupling between neighbors (Liu and Fetcho 1999; Burgess and Granato 2007). Our analysis partitioned behavior into periods of acceleration and deceleration, characterizing over 90% of collective motion to resolve the timing of individual responses within the social network (Peterson et al. 2024). We found that the LLS significantly reduced response latency, but exclusively during collective accelerations. In these periods, fish with intact flow sensing exhibited a *∼*33% lower latency (*0.175 ± 0.097* s) compared to those with compromised sensing (*0.262 ± 0.125* s; $P_\mathrm{accel}=0.025$; Table 2; Fig. 4A). This sensory enhancement was particularly evident during low-magnitude accelerations ($m_{\mathrm{low}}$, $P_\mathrm{accel}=0.006$), where the LLS appeared to facilitate coordination when kinematic signals are less intense. Conversely, response latency was statistically indistinguishable between groups during decelerations, indicating that flow sensing offered no measurable advantage when the school was slowing down (Fig. 4A). Across all behavioral states and sensory conditions, interfish distance (*d*) acted as a fundamental constraint on coordination. Pairwise comparisons showed that latency increased and correlation strength decreased significantly as spacing widened (*γ*, *P < 0.001*; *|τ |*, *P < 0.001*; Figs. 3 and 4C and F). These kinematic results are consistent with our predictions and demonstrate that while behavioral magnitude sets the overall baseline of coordination, the lateral line acts as a specialized, rapid channel for information transfer during acceleration.

**Table 1:** Table of symbols

Symbol	Quantity
*C*	Algebraic connectivity
*D*	Degree
d	Pairwise minimum distance
*E*	Global efficiency
*H*	Network heterogeneity
LLS	Lateral-line system
*M*	Adjacency matrix of MI values
MI	Mutual information
*m*	Behavioral period magnitude
NND	Nearest-neighbor distance
*p(a)*	Marginal probability distribution
*p(a, b)*	Joint probability distribution
*s*	Speed of a fish
*u*	Flow speed detected by a fish
*γ*	Pairwise cross-correlation coefficient
*Γ*	Segment average *γ*
$\gamma _\mathrm{flow}$	Flow cross-correlation coefficient
*ρ*	Polarization parameter
*τ*	Pairwise cross-correlation response latency
*T*	Segment average *τ*
$\tau _\mathrm{flow}$	Flow cross-correlation response latency

**Table 2:** Significant fixed effects (*P < 0.05*): pairwise correlation (*γ*) and latency (*τ*)

Subtest	Measure	Independent variable	Estimate	SE	*P*
**Acceleration period**					
*-*	*γ*	(Intercept)	0.8736	0.0036	0.0000
*-*	*γ*	*d*	−0.0007	0.0000	0.0000
*-*	*τ*	(Intercept)	3.6503	0.4749	0.0013
*-*	*τ*	*LLS*	2.2761	0.6849	0.0246
*-*	*τ*	*d*	0.0551	0.0021	0.0000
$m_{{\rm low}}$	*γ*	(Intercept)	0.8646	0.0045	0.0000
	*γ*	*d*	−0.0006	0.0000	0.0000
	*τ*	(Intercept)	4.4808	0.6501	0.0000
	*τ*	*LLS*	2.5140	0.8923	0.0063
	*τ*	*d*	0.0643	0.0035	0.0000
$m_{{\rm high}}$	*γ*	(Intercept)	0.8810	0.0035	0.0000
	*γ*	*d*	−0.0008	0.0000	0.0000
	*τ*	(Intercept)	3.0495	0.4085	0.0019
	*τ*	*d*	0.0462	0.0024	0.0000
LLS present	*γ*	(Intercept)	0.8725	0.0057	0.0000
	*γ*	*m*	0.0111	0.0049	0.0259
	*γ*	*d*	−0.0009	0.0000	0.0000
	*τ*	(Intercept)	4.8506	0.6532	0.0017
	*τ*	*m*	−1.8820	0.6092	0.0028
	*τ*	*d*	0.0483	0.0025	0.0000
LLS absent	*γ*	(Intercept)	0.8479	0.0035	0.0000
	*γ*	*m*	0.0200	0.0052	0.0003
	*γ*	*d*	−0.0005	0.0000	0.0000
	*τ*	(Intercept)	7.1027	0.5563	0.0000
	*τ*	*m*	−3.1408	0.8285	0.0003
	*τ*	*d*	0.0610	0.0033	0.0000
**Deceleration period**					
*-*	*γ*	(Intercept)	0.8912	0.0057	0.0000
*-*	*γ*	*d*	−0.0005	0.0000	0.0000
*-*	*τ*	*d*	0.0646	0.0019	0.0000
$m_{{\rm low}}$	*γ*	(Intercept)	0.8755	0.0064	0.0000
	*γ*	*d*	−0.0005	0.0000	0.0000
	*τ*	*d*	0.0761	0.0036	0.0000
$m_{{\rm high}}$	*γ*	(Intercept)	0.9011	0.0031	0.0000
	*γ*	*d*	−0.0005	0.0000	0.0000
	*τ*	(Intercept)	2.0214	0.4977	0.0379
	*τ*	*d*	0.0553	0.0020	0.0000
LLS present	*γ*	(Intercept)	0.8758	0.0049	0.0000
	*γ*	*m*	0.0255	0.0056	0.0000
	*γ*	*d*	−0.0005	0.0000	0.0000
	*τ*	(Intercept)	4.0683	0.8128	0.0137
	*τ*	*m*	−1.9903	0.5889	0.0012
	*τ*	*d*	0.0513	0.0021	0.0000
LLS absent	*γ*	(Intercept)	0.8696	0.0044	0.0000
	*γ*	*m*	0.0299	0.0069	0.0002
	*γ*	*d*	−0.0005	0.0000	0.0000
	*τ*	(Intercept)	6.0855	1.1270	0.0148
	*τ*	*m*	−3.4364	1.4276	0.0198
	*τ*	*d*	0.0770	0.0033	0.0000

### Information transmission and network topology

**Fig. 5:**
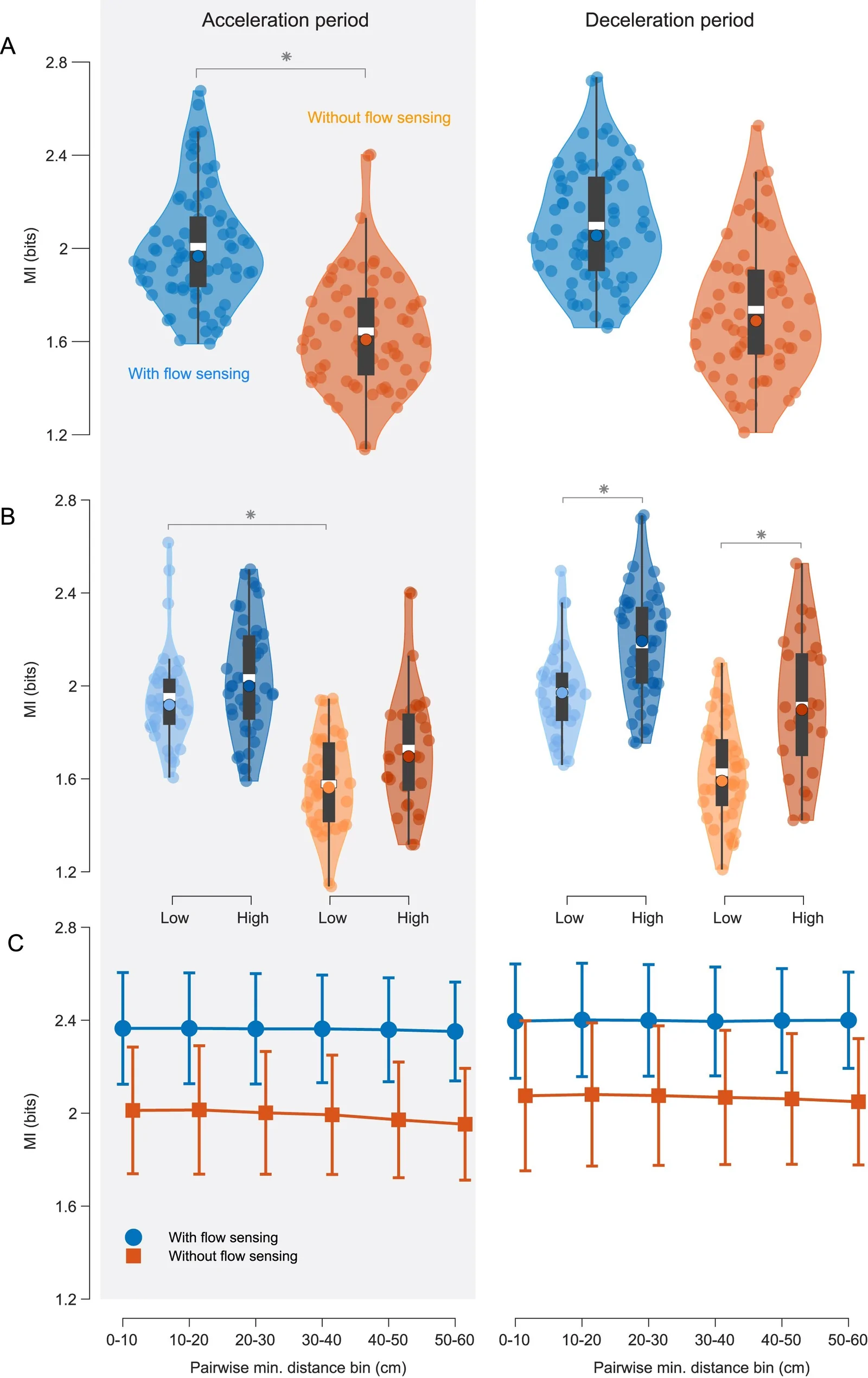
Average MI values shared between schooling rummy-nose tetras. (A–B) Violin plots depict the probability distributions of schools with (in blue) and without (in orange) flow sensing, with segment averages (transparent colored dots) and box plots that depict the first and third quartiles of the data, the median (solid colored dot), and the mean value (white bar); whiskers show the range of the data. Comparisons are shown for (A) all accelerations and decelerations and (B) when parsed by the magnitude of those events. Connecting horizontal lines with an asterisk depict significant relationships between groups of data. (C) Variation of MI with the distance between pairs of fish, binned by distance (10 cm, circle and square markers). For display, we truncated distance bins to 60 cm. Error bars depict *1±* standard deviation.

**Fig. 6:**
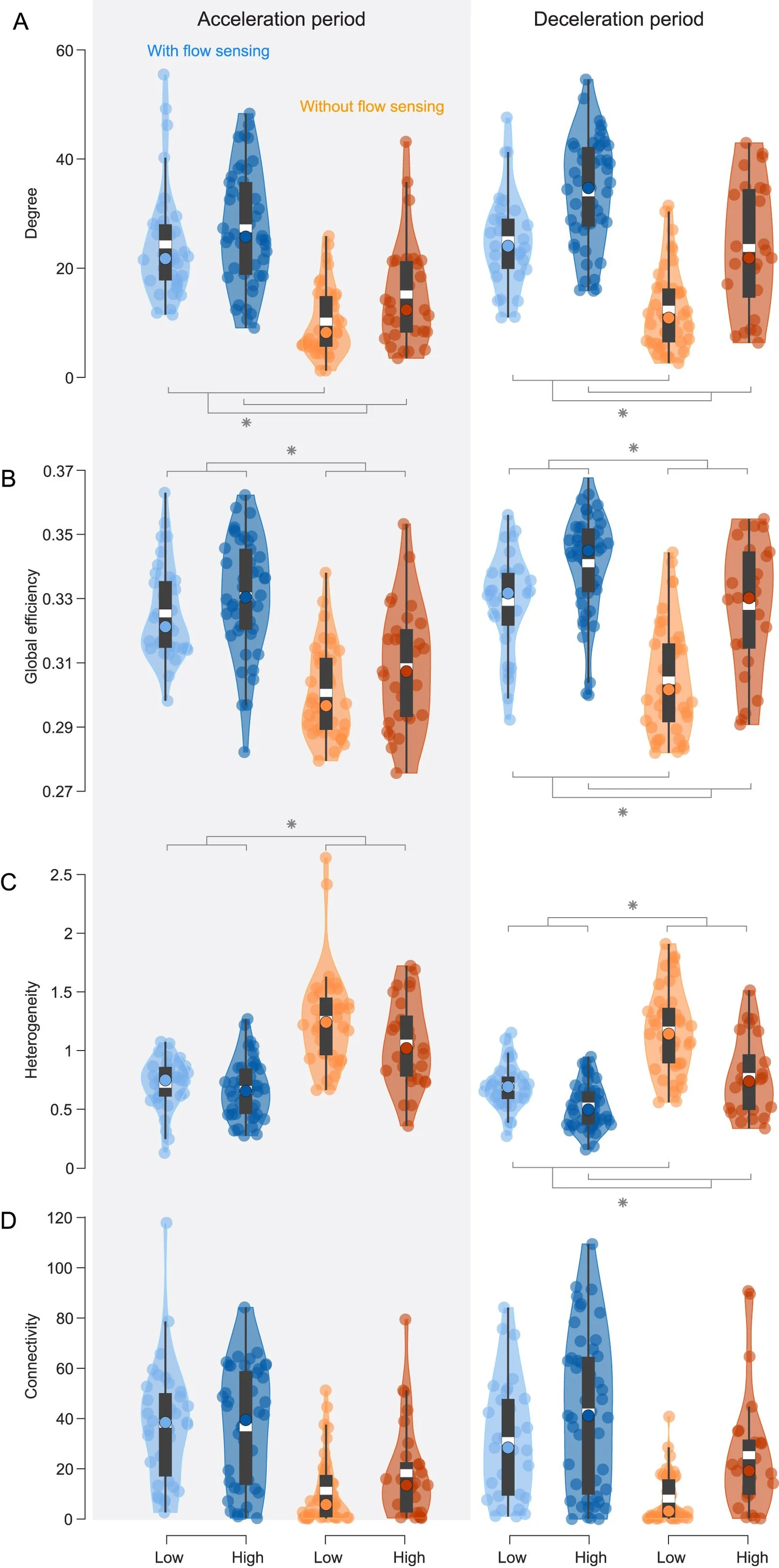
Network properties of schooling rummy-nose tetras. (A–D) Violin plots depict the probability distributions of segment averages (transparent colored dots) of (A) degree, (B) global efficiency, (C) network heterogeneity, and (D) algebraic connectivity within behavioral periods for schools that sense flow (in blue) and those that cannot (in orange). Light and dark shades of each color depict “low” and “high” magnitude, respectively, of accelerations and decelerations. A box plot within each violin plot depicts the first and third quartiles of the data, the median (solid colored dot), and the mean value (white bar); whiskers show the range of the data. Connecting horizontal lines with an asterisk depict significant relationships between groups of data. Here, connecting horizontal lines above the violin plots depict significance between pooled magnitude behaviors of schools with, and without, flow sensing. Lines below depict significance between “low” and “high” magnitude behaviors within treatment groups.

MI serves as a robust pairwise measure of shared information, quantifying how the kinematic state of one fish reduces uncertainty regarding its neighbor (Peterson et al. 2024). Flow sensing significantly enhanced MI during acceleration ($P_\mathrm{accel}=0.043$), particularly during low-magnitude movements where MI was higher in untreated schools than in treated ones ($P_\mathrm{accel} < 0.027$; Table 3; Fig. 5B). During deceleration, however, MI was largely determined by behavioral magnitude, rather than sensory modality. Both intact and compromised schools exhibited significantly lower MI during low-magnitude slowing compared to high-magnitude deceleration (LLS present, $P_\mathrm{decel} < 0.001$; LLS absent, $P_\mathrm{decel} < 0.004$; Fig. 5B). The spatial constraints of information sharing remained evident across all conditions, with pairwise distance exerting a consistently strong negative effect on MI (*P < 0.001*; Fig. 5A and C). The degradation of flow sensing also fundamentally altered the emergent architecture of the school’s communication network. Global efficiency (*E*), which characterizes the speed of information propagation through the group, was significantly lower in schools without LLS function during both acceleration and deceleration ($P_\mathrm{accel}=0.021, P_\mathrm{decel}=0.040$; Table 4; Fig. 6B). Furthermore, network heterogeneity (*H*) increased in the absence of flow sensing ($P_\mathrm{accel}=0.036, P_\mathrm{decel}=0.042$), suggesting a less uniform and more fragmented communication structure (Fig. 6C). While fish relying solely on vision may maintain local links, the resulting network is more variable and less efficient, likely due to neighbors being visually occluded. This confirms our prediction that the loss of flow sensing impedes the effective propagation of information throughout a school. The LLS therefore enhances not only pairwise coordination but also the overall cohesion and efficiency of the information network underlying collective behavior.

**Table 3:** Significant fixed effects (*P < 0.05*): pairwise mutual information

Subtest	Measure	Independent variable	Estimate	SE	*P*
Acceleration period					
*-*	MI	(Intercept)	2.3418	0.0876	0.0000
*-*	MI	*LLS*	−0.3628	0.1240	0.0431
*-*	MI	*d*	−0.0009	0.0000	0.0000
$m_{\mathrm{low}}$	MI	(Intercept)	2.3176	0.0752	0.0000
	MI	*LLS*	−0.3658	0.1063	0.0265
	MI	*d*	−0.0007	0.0000	0.0000
$m_{\mathrm{high}}$	MI	(Intercept)	2.3640	0.0946	0.0000
	MI	*d*	−0.0011	0.0000	0.0000
LLS present	MI	(Intercept)	2.3272	0.0618	0.0003
	MI	*d*	−0.0011	0.0001	0.0000
LLS absent	MI	(Intercept)	1.9579	0.1057	0.0027
	MI	*d*	−0.0008	0.0000	0.0000
**Deceleration period**					
*-*	MI	(Intercept)	2.3873	0.0967	0.0000
*-*	MI	*d*	−0.0009	0.0000	0.0000
$m_{\mathrm{low}}$	MI	(Intercept)	2.3311	0.0896	0.0000
	MI	*d*	−0.0009	0.0000	0.0000
$m_{\mathrm{high}}$	MI	(Intercept)	2.4233	0.0870	0.0000
	MI	*d*	−0.0009	0.0000	0.0000
LLS present	MI	(Intercept)	2.3327	0.0569	0.0002
	MI	*m*	0.0932	0.0262	0.0007
	MI	*d*	−0.0010	0.0000	0.0000
LLS absent	MI	(Intercept)	1.9993	0.1109	0.0028
	MI	*m*	0.0967	0.0321	0.0038
	MI	*d*	−0.0009	0.0000	0.0000

**Table 4:** Significant fixed effects (*P < 0.05*): network metrics

Measure	Independent variable	Estimate	SE	*P*
**Acceleration period**				
*D*	(Intercept)	3.1036	0.2430	0.0002
*D*	*m*	0.1554	0.0765	0.0440
*E*	(Intercept)	0.3259	0.0048	0.0000
*E*	*LLS*	−0.0244	0.0065	0.0208
*H*	(Intercept)	−0.4036	0.1297	0.0313
*H*	*LLS*	0.5574	0.1786	0.0357
*C*	(Intercept)	3.2685	0.7593	0.0121
*ρ*	(Intercept)	0.5145	0.0232	0.0000
*ρ*	*m*	0.0556	0.0222	0.0134
${\it s}$	(Intercept)	2.1471	0.1058	0.0000
${\it s}$	*m*	0.0667	0.0241	0.0065
**Deceleration period**				
*D*	(Intercept)	3.1520	0.1962	0.0000
*D*	*m*	0.3429	0.0769	0.0000
*E*	(Intercept)	0.3280	0.0050	0.0000
*E*	*LLS*	−0.0205	0.0068	0.0402
*E*	*m*	0.0135	0.0029	0.0000
*H*	(Intercept)	−0.4234	0.1214	0.0201
*H*	*LLS*	0.4878	0.1655	0.0424
*H*	*m*	−0.2961	0.0595	0.0000
*ρ*	(Intercept)	0.5879	0.0252	0.0000
*ρ*	*m*	0.1042	0.0229	0.0000
${\it s}$	(Intercept)	2.1302	0.1097	0.0000
${\it s}$	*m*	0.0748	0.0264	0.0053

### The role of flow sensing as a fast sensory pathway

**Fig. 7:**
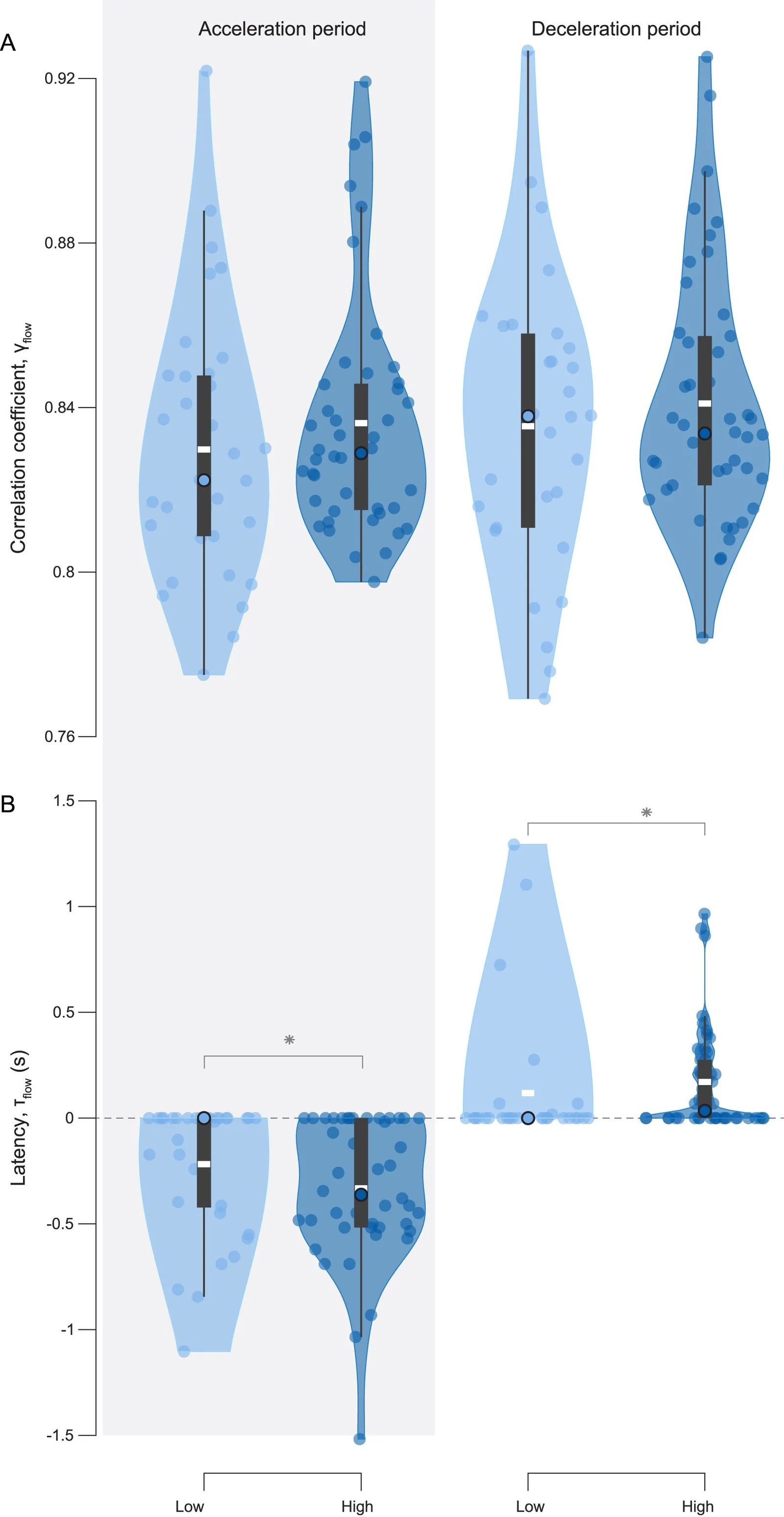
The results of a signed cross-correlation analysis evaluating the response of rummy-nose tetras to hydrodynamic stimuli during periods of collective acceleration and deceleration. (A and B) Violin plots depict the probability distributions of segment averages (transparent colored dots) of $\gamma _\mathrm{flow}$ and $\tau _\mathrm{flow}$ within behavioral periods for schools with the ability to sense flow, subdivided by the magnitude of the behavior period. Light blue and dark blue shades depict “low” and “high” magnitude segments, respectively. A box plot within each violin plot depicts the first and third quartiles of the data, the median (solid colored dot), and the mean value (white bar); whiskers show the range of the data. Connecting horizontal lines with an asterisk depict significant relationships between groups of data.

The divergent role of flow sensing during acceleration and deceleration is supported by the hydrodynamic cues available to the fish. By simulating flow velocities encountered during schooling (Zhou et al. 2025), we found that fish speed tended to lag behind detected flow stimuli during accelerations, indicated by a negative response latency (*-0.384 ± 0.355* s; Fig. 7B). This negative latency indicates that accelerating fish responded to the hydrodynamic signals generated by their neighbors’ movements. In contrast, latencies during deceleration were overwhelmingly positive (*0.278 ± 0.257* s), meaning changes in swimming speed preceded the detected flow signals (Fig. 7, Table 5). Therefore, hydrodynamic cues during these slowing periods appeared to be a consequence of a fish’s own motion rather than the flow generated by a neighbor. Mechanistically, acceleration requires the generation of high-velocity wakes and thrust-producing vortices that provide strong, localized cues to neighbors (Drucker and Lauder 1999; Wise et al. 2018). These signals likely stimulate both superficial and canal neuromasts, providing a compounded mechanosensory cue that triggers rapid coordination (Engelmann et al. 2002; Klein and Bleckmann 2011; Carrillo et al. 2019). Because visual processing is comparatively slow and subject to occlusion, the LLS provides a critical pathway for matching rapid neighbor movements (Davidson et al. 2021; Tunstrøm et al. 2013). Rather than serving as a redundant channel to vision, the lateral line acts as a specialized, fast sensory pathway that ensures tight temporal coupling precisely when collective demands are greatest. In summary, the lateral line provides a rapid channel of information that enhances the cohesion and efficiency of social networks in schools during the behavioral contexts that demand it most.

**Table 5:** Significant fixed effects (*P < 0.05*): flow-aligned $\gamma _{\mathrm{flow}}$ and $\tau _{\mathrm{flow}}$

Subtest	Measure	Independent variable	Estimate	SE	*P*
**Acceleration period**					
*-*	$\tau _{\mathrm{flow}}$	(Intercept)	−1.0785	0.1903	0.0021
*-*	$\tau _{\mathrm{flow}}$	*m*	−0.3450	0.1214	0.0052
**Deceleration period**					
*-*	$\tau _{\mathrm{flow}}$	(Intercept)	0.6107	0.1442	0.0059
*-*	$\tau _{\mathrm{flow}}$	*m*	0.4931	0.1147	0.0000

## Conclusions

The present study examined how flow sensing contributes to the coordination and communication of schooling fish by combining hydrodynamic modeling with a detailed kinematic and network analysis of schools with and without lateral-line function. Our results reveal a sensory asymmetry in the control of schooling: flow sensing selectively enhanced coordination of changes in speed during collective acceleration, when fish followed hydrodynamic cues generated by neighbors. Flow sensing appeared to offer no measurable advantage for the coordination of schooling fish during deceleration, when changes in fish swimming speeds preceded the flow signals they detected. This difference between accelerations and decelerations was evident across multiple levels of analysis, from pairwise response latency and correlation coefficients, to MI. The efficiency and uniformity of the school’s communication network were enhanced by flow sensing throughout both behavioral periods. Inter-fish distance imposed a strong constraint on all metrics of coordination and communication, underscoring the fundamentally local nature of sensory interactions in schools. These findings indicate that the lateral line does not serve as a redundant sensory channel alongside vision, but rather as a fast, locomotor-dependent pathway that tightens temporal coupling between neighbors during the collective behaviors that generate the strongest hydrodynamic signals. More broadly, our results demonstrate that the emergent network properties of a school arise from the sensorimotor capabilities of its members, and that resolving the transient dynamics of collective behavior is necessary to reveal the mechanistic contributions of individual sensory systems.

## Data Availability

Code and data are available for download from the Dryad repository: https://doi.org/10.5061/dryad.gxd25482b.

## Notes

From the symposium “Animal collective movement in complex environments” presented at the annual meeting of the Society for Integrative and Comparative Biology, January 3–7th, 2026.
